# Dome-arrayed chitosan/PVA hydrogel-based solar evaporator for steam generation

**DOI:** 10.1038/s41598-022-08589-z

**Published:** 2022-03-15

**Authors:** Minyue Zhu, Xiaojie Liu, Yanpei Tian, Andrew Caratenuto, Fangqi Chen, Yi Zheng

**Affiliations:** Department of Mechanical and Industrial Engineering, Northeastern University, Boston, MA 02115 USA

**Keywords:** Energy harvesting, Renewable energy

## Abstract

Water evaporation systems with solar energy as the primary driving energy have received extensive attention in recent years. This work studies the preparation method and performance of hydrogel evaporators using chitosan and polyvinyl alcohol (PVA) as a framework and carbon nanoparticles (CNPs) as the photothermal material. The evaporation rate of CPC (chitosan/PVA and CNPs) hydrogel obtained reaches 2.28 kg m^−2^ h^−1^. Simultaneously, a three-dimensional structure is designed based on the two-dimensional double-layer evaporation system in this study. An evaporator with a tiny-pool structure and a hydrogel with a dome-arrayed structure is designed. These two structures achieve highly efficient evaporation rates of 2.28 kg m^−2^ h^−1^ and 3.80 kg m^−2^ h^−1^, respectively. These optimized designs improve the evaporation rate of the overall system by ~ 66.7%. The developed evaporation devices provide a promising pathway for developing the double-layer evaporators, which promote the new development of water purification with a solar-driven evaporation system.

## Introduction

Water is the most precious resource for maintaining human survival and economic development^1,2^. However, the amount of fresh water on the earth surface is limited and not capable of meeting the increasing demand. At the same time, the ocean covers more than 70% earth’s surface area^2^. Thus, the problem of converting seawater into fresh water for human utilization is a vital topic that many researchers are working on to aid the sustainability of human societies.

Traditional water evaporation factories are based on petroleum. This method causes significant wastewater pollution and greenhouse gas emissions. In order to reduce environmental pollution and monetary cost, researchers are driven to utilize a renewable source as the driving energy for evaporation^3^. The solar-driven steam generation system is a method that can easily convert seawater to freshwater based on abundant, renewable and clean solar energy^4^. Solar distillation mimics the natural water cycle, in which the sun heats seawater to the point of evaporation^5^. After evaporation, the water vapor condenses on a cooler surface. In general, solar-driven water steam generation systems can be divided into two types. One is using photovoltaic (PV) cells which convert solar energy to electricity to power the evaporation process. The other one is utilizing the solar thermal energy directly as the driving energy for evaporation. The PV desalination system includes reverse osmosis (RO) and electrodialysis (ED). The solar thermal system includes multi-stage flash (MSF), multi-effect distillation (MED), vapor compression (VC), freeze desalination (FD) and membrane distillation (MD)^6^.

The main purpose of the current research of solar thermal desalination system is to improve the photothermal efficiency and the quality of distilled water after purification. Researchers are committed to improving the heat absorption rate of light absorber, the ability to trap sunlight on the gas–liquid surface, and the usable surface area of light absorber per unit projection area, as these are key factors in creating a highly effective desalination system.

The double layer system consists of a light absorber with broadband absorption performance, a thermal insulation barrier and a hydrophilic water channel to transfer the seawater to the light absorber. This system with an excellent evaporation efficiency has been vastly studied. In 2013, Omara et al.^7^ designed a two-tier desalination distillation system using an evacuated solar water heater, Jut geotextile and a solar still. This double-layered square wick (DLSW) solar still increased water productivity by 114%. The average daily efficiency of the DLSW was 71.5% and the productivity of distilled water increased by 215% if hot brackish water was fed at night. Lee et al.^8^ proposed a super hydrophilic thermally insulated microporous membrane composed of carbonized sucrose and polydimethylsiloxane as an efficient solar evaporator, which reached a purified water production rate of 1.28 kg m^−2^ h^−1^. In their study, solar energy is used as the only driving energy. The photothermal conversion absorber receives solar energy from sunlight and heats the water inside the porous structure^9^. It requires broadband spectrum absorbing performance and high photothermal conversion efficiency.

The main purpose of the heat insulation layer is to trap the thermal energy obtained by the light absorber on the gas–liquid interface. This prevents heat diffusion to the bulk water, thus enhancing the efficiency. As such, the heat barrier material should own low thermal conductivity and hydrophobic properties, which minimize the conductive heat loss in the transport process. The combination of the thermal barrier and a hydrophilic water channel can create a two-dimensional (2D) water transfer system. Shrinking the waterway can effectively reduce the downward heat diffusion to bulk water, improving the evaporation efficiency. Previously employed insulation materials include wood^10,11^, air-laid paper^12^, macro-porous silica substrate^13^ and polystyrene foam^14^. The 2D water supply employs hydrophilic intermediary materials such as cellulose^15^, cotton and silk fabrics^16^, vertically oriented graphene structures^17^ and air‐laid paper^18^ for water delivery. In this study, Polyvinyl chloride (PVC) foam is applied as the heat insulation material in the bottom layer. As a closed-cell material, PVC foam has the ability to seal out moisture and has high hardness. As a thermoplastic, PVC performs an excellent performance in thermal insulation. These properties make PVC foam a very suitable material for the heat insulation barrier. At the same time, a cotton wipe is used with high water absorption as the water pipeline between the hydrogel and the bulk water. An adequate and continuous water supply contributes greatly to the high evaporation performance. Its structure affects the water flow’s direction to the absorbing layer. which indirectly affects the evaporation efficiency.

Hydrogels, which are three-dimensional (3D) porous polymeric networks consisting of hydrophilic polymer chains, have been widely employed in various areas such as inclusive biosensors^19^, hygiene products^20^, contact lenses, drug delivery^21^, and cell culture^22^. In recent years, hydrogels have shown incredible evaporation performance and become a promising functional material in this field. Hydrogels cooperate well with various types of nanoparticles to improve the photothermal conversion ability, making it suitable for solar-driven water evaporation system. As a porous structure, hydrogels have a large internal volume and surface area, which can continuously absorb water in the process of evaporation and provides sufficient gas–liquid contact to realize an efficient evaporation performance. In this work, the design and optimization of evaporation system and CPC hydrogel evaporator structure is conducted. A tiny-pool structure and dome-arrayed hydrogel is designed. The hydrogel water absorption and light absorption and the overall evaporation rate of the system are measured to demonstrate the feasibility of the system. PVA is a water-soluble polymer with excellent chemical stability, film-forming ability and high hydrophilicity^23^. Chitosan is the chitin of shrimps and other crustaceans that has been treated with an alkaline substance. When these two materials cross-linked, a porous hydrogel framework with high flexibility, toughness and water-absorbing quality will be formed. As a carbon-based material, CNPs have extraordinary thermal conductivity and high stability. Therefore, it is used as the light absorbing material of this hydrogel to maximize the solar energy gain.

**Figure 1:**
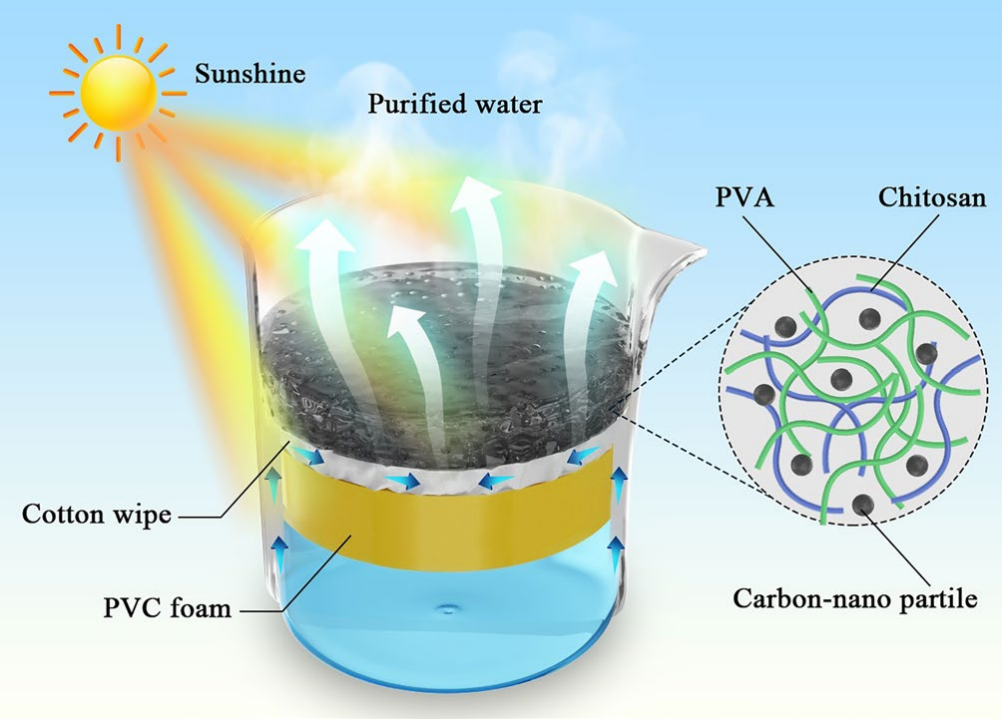
Schematic illustration of regular double-layer evaporation system.

In this study, a photothermal, macro-porous double-layer solar evaporation system is reported with the schematic structure shown in Fig. 1. A chitosan/PVA/CNPs (CPC) hydrogel is introduced using chitosan and PVA as the structure network of the hydrogel and CNPs distributed into the chitosan/PVA matrix as a photothermal material.

### Dome-arrayed hydrogel

**Figure 2:**
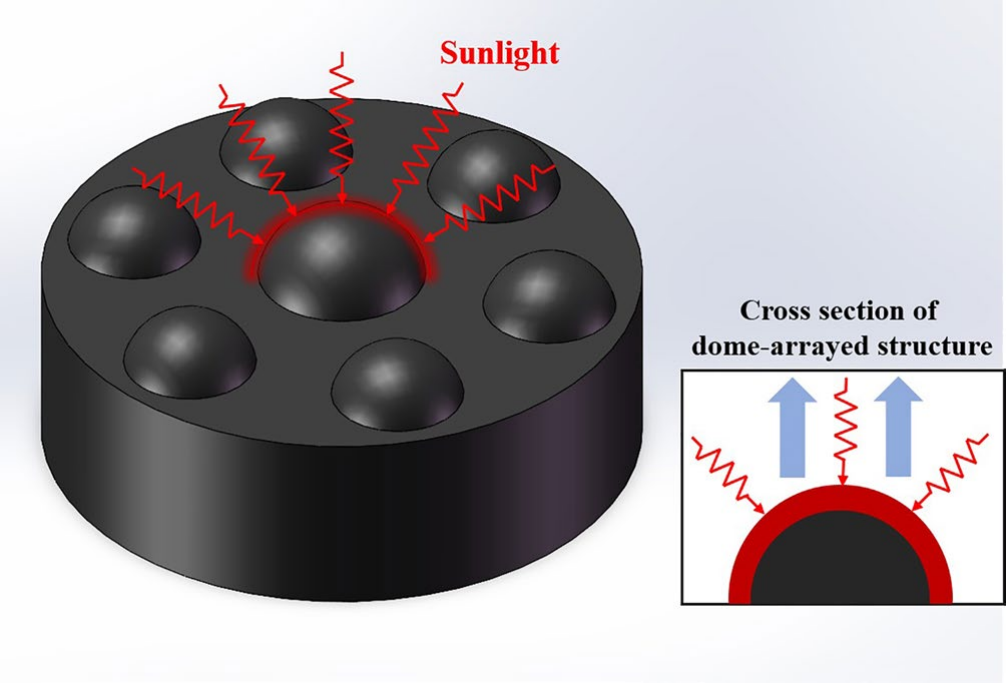
Optimized dome-arrayed hydrogel with hemispherical structures.

To increase the evaporation rate and light absorption of the hydrogel, the original planar hydrogel is optimized. A dome-arrayed hydrogel with several hemispherical projections on its surface is designed, as shown in Fig. 2.

In the practical application, the surface area of the evaporator plays a decisive role in the evaporation rate. The larger surface area can effectively enhance the convective heat dissipated on the surface of the evaporator, thus increasing the evaporation rate. The hemispherical structure can create more evaporate surface area without changing the project area. This dome-arrayed structure therefore dramatically increases the light absorption of the hydrogel, as well as the evaporation area, thus increasing the evaporation rate.

After evaluating the shading impact to determine the geometry which best promotes photothermal absorption, seven hemisphere units with a radius of 2 mm are added to a cylindrical hydrogel with a diameter of 70 mm. The hydrogel is made with custom silicone molds, and the surface is polished. In the subsequent experiments, the evaporation performance of a single hemisphere unit is tested, and the experimental results prove that the structure could indeed improve the evaporation performance.

## Materials and methods

### Materials

PVA, molecular weight of 85,000–124,000, 99 + % hydrolyzed. Chitosan, medium molecular weight, from Sigma-Aldrich. Glutaraldehyde solution, 50 wt. % in H2O, from Sigma-Aldrich.

### Methods

#### Fabrication of chitosan/PVA-CNP hydrogel

**Figure 3:**
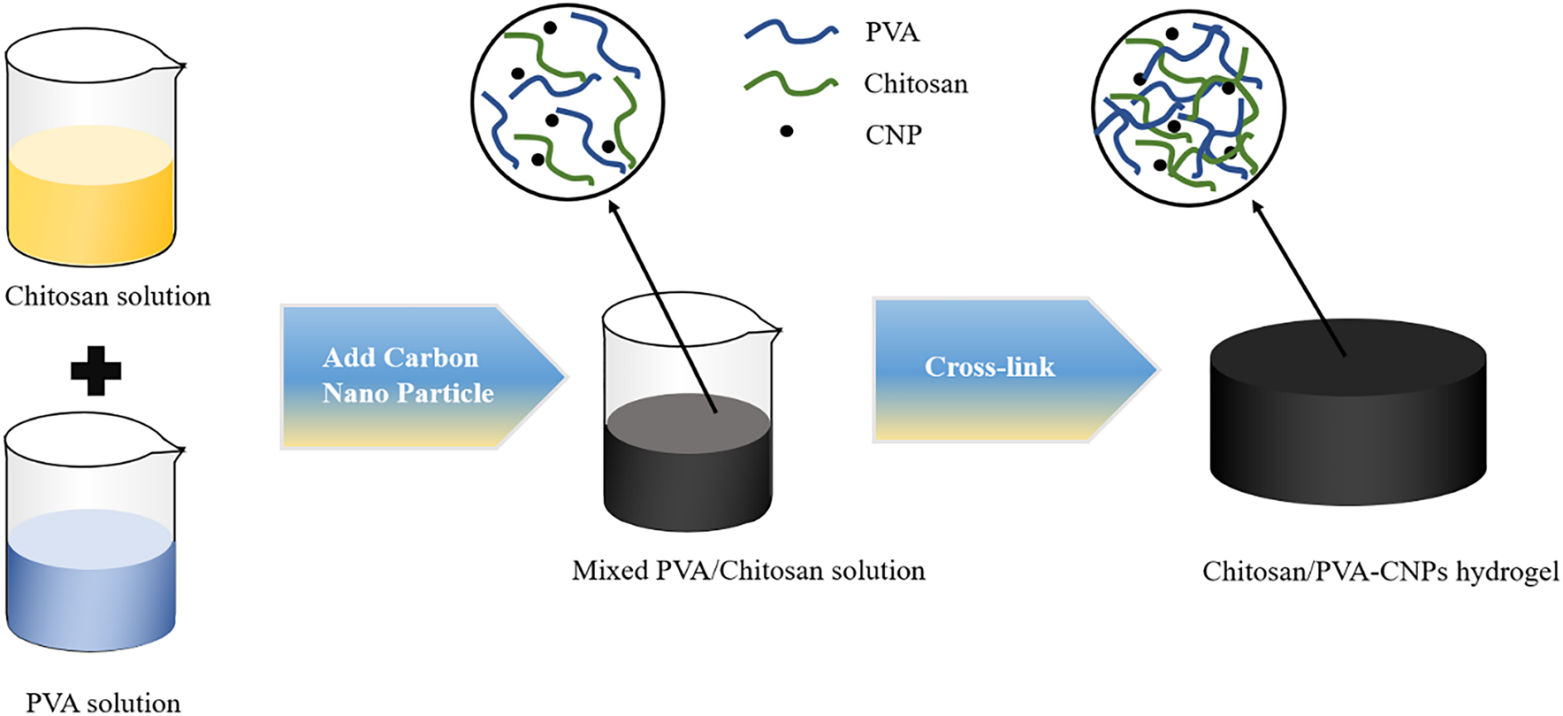
Fabrication process of CPC hydrogel.

The general schematic of the hydrogel fabrication process is shown in Fig. 3. The sample is a cylinder with a diameter of 70 mm and a thickness of 10 mm. First, 1 g of chitosan powder is dissolved in 50 ml 1% (v/v) acetic acid solution and stirred with a magnetic stirrer at 40 °C for 6 h until completely dissolved. Then, we dissolve 4 g of PVA powder in 40 ml of deionized water and stir with a magnetic stirrer at 90 °C for 3 h until completely dissolved. Next, the PVA solution and chitosan solution are mixed and 0.5 g of CNPs powder is added. The mixture is evenly mixed with a ultrasonication device. This constitutes preparation of the pre-solution of hydrogel.

Then, 625 μl glutaraldehyde solution is added into the mixed Chitosan/PVA-CNP solution as cross-linker. The solution is molded and pre-frozen in refrigerator at − 20 °C for 24 h. A lyophilizer is used to freeze dry the sample for 24 h until it is totally dehydrated. Finally, the obtained hydrogel sample is fully swollen for testing.

#### Surface treatment

The surface treatment removes the clogged structure of the material surface after the lyophilization treatment, allowing the hydrophobic pipe in direct contact with the air and improving the evaporation efficiency.

An electric grinder is used to polish the upper and lower surfaces of the dry CPC hydrogel, with a longitudinal grinding depth of 0.5 mm on each side. After grinding, the hydrogel is soaked in water, and the evaporation efficiency is tested after fully absorbing water.

## Results and discussion

### Characterization

The morphology and microstructure of the samples are examined by scanning electron microscopy (SEM, Supra 25) under an acceleration voltage of 5 kV. The contact angle is characterized by the SINDIN SDC-350 contact angle meter. The reflectance spectra from 0.3 to 2.5 µm are measured by a Jasco V770 spectrophotometer equipped with a Jasco ISN-923 integrating sphere with a fixed angle of 6°. Infrared images are taken using FLIR A655C thermal camera with a 25° lens at a resolution of 640 × 480.

**Figure 4:**
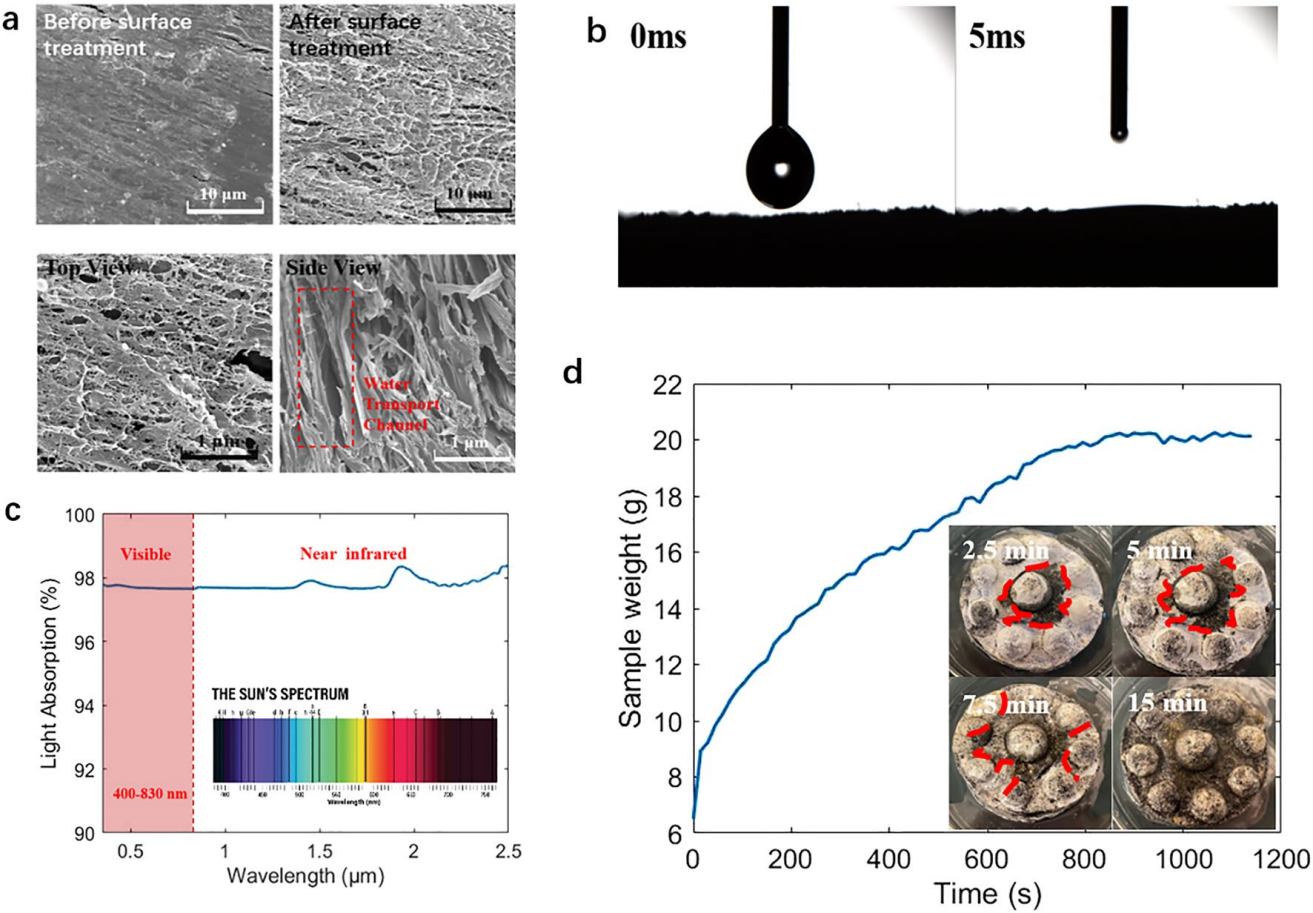
(**a**) SEM images of CPC hydrogel sample. (**b**) Contact angel of CPC hydrogel sample. (**c**) Light absorption of a CPC hydrogel sample in visible and infrared ranges. (**d**) Wetting process of hydrogel with dome-arrayed structure and sample weight curve.

The CPC hydrogel evaporator presents a fluffy structure similar to a sponge. After fully soaking in water, it presents a uniform water absorption performance. Each unit within the structure is consistent in terms of water-absorbing rate and amount. The sample is soft and has a good toughness, as it can be squeezed and pulled back to the original shape. The SEM images of the CPC hydrogel are shown in Fig. 4a. The CPC hydrogel has a uniform internal water channel, making it a suitable material for a near-ideal water evaporator.

As the water evaporates, the CPC hydrogel absorbs sunlight and converts it into thermal energy mainly due to the high photothermal absorption of the added CNPs. As such, the spectral absorptivity of the material plays a crucial role in evaporation performance; when spectral absorption is high, more sunlight can be absorbed, resulting in more thermal energy available for the photothermal conversion.

A Jasco V770 spectrophotometer is used to characterize the spectral transmittance and reflectance of CPC hydrogel sample. Its light absorption is calculated by $A=1-T-R$, here *A* represents absorptance, *T* represents transmittance, and *R* represents reflectance. As shown in Fig. 4c, the CPC hydrogel sample in the wavelength range of 0.3–2.5 µm achieves an average light absorption rate of up to 98%. It maintains a light absorption rate of nearly 98% over the entire visible wavelength range (0.4–0.83 µm). The CNPs play a vital role in the process of light absorption and can absorb and convert the majority of the incident energy, which highly enhances the evaporation efficiency of this hydrogel. Therefore, it can be concluded that the hydrogel prepared by adding CNPs into chitosan/PVA framework has a great light absorption rate and is suitable to be the light-absorbing material of the evaporator.

Water absorption capacity is also an essential factor affecting the evaporation efficiency of materials. The water absorption of a material controls the quantity of water available for evaporation as a result of its ability to resupply water to the evaporation. Superabsorbent materials can quickly absorb water from the water transport layer when their internal water evaporation keeps its internal water sufficient and continues to evaporation. If the material is not absorbent enough, it will not take in enough water from the absorbent layer to sustain subsequent evaporation when most of the water in the material is evaporated. This feature would render the material dry, gradually terminating evaporation and significantly reducing efficiency. Due to the porous structure of chitosan/PVA framework, it is expected to have high water absorption. This expectation is proven by measuring the contact angle and the process of water absorption.

The contact angle is the angle formed between the liquid–vapor interface and the solid surface when the liquid drops on the solid surface, indicating the object's hydrophilicity and wettability. Generally speaking, for a solid that absorbs water, the dynamic phenomenon that the contact angle changes when the liquid drops on the surface, the range is gradually reduced from the maximum contact angle to the minimum contact angle, reflecting the water absorption speed of the material. In this experiment, a water droplet is deposited on the thoroughly soaked hydrogel sample, and images are captured with a high-speed camera illustrating the change of contact angle in the absorption process.

It can be found that the CPC hydrogel absorbed the water by capturing the images as soon as it dropped on the surface which showed in Fig. 4b. The contact angle drops to 0 degrees within 5 ms, and the absorption process can hardly be observed. This phenomenon indicates that the surface of the treated hydrogel is completely hydrophilic and has excellent water absorption. This experimental result proves that this sample can continuously absorb water from the water transport layer and maintain the continuous process of evaporation in the evaporation process.

The water absorption test of the hydrogel sample records the water absorption process of the dehydrated sample. In this experiment, a dry sample is tested with a hemispherical structure. To facilitate the observation of moisture infiltration, only the lower surface in contact with the water is polished. A dehydrated sample is dipped into a glass dish filled with water, and its weight is recorded every 15 s until stable, at which point it is considered to be thoroughly wet.

As shown in Fig. 4d, the sample is gradually wetted from the center, with the hemisphere structure at the top being the last part to be saturated. The total soaking time is about 15 min. The dry sample weighs 6.5 g, and it experiences a gradual increase in weight before reaching to a converging value, that indicates the entirely wet sample weighs about 20.2 g. The swelling rate is calculated of the sample by $ESR={W}_{S}/{W}_{D}$, here *ESR* represents the equilibrium swelling rate. *W*_*S*_ represents the mass of the hydrogel after swelling equilibrium, and *W*_*D*_ represents the mass of the dry hydrogel.

The ESR of this sample is 3.10, which shows the sample has an excellent internal wettability. According to the above experimental results, it is fully supported that this sample is suitable as the light absorber material within a desalination system because it can continuously absorb water from the water transport layer and maintain continuous evaporation.

### Evaporation experiments

**Figure 5:**
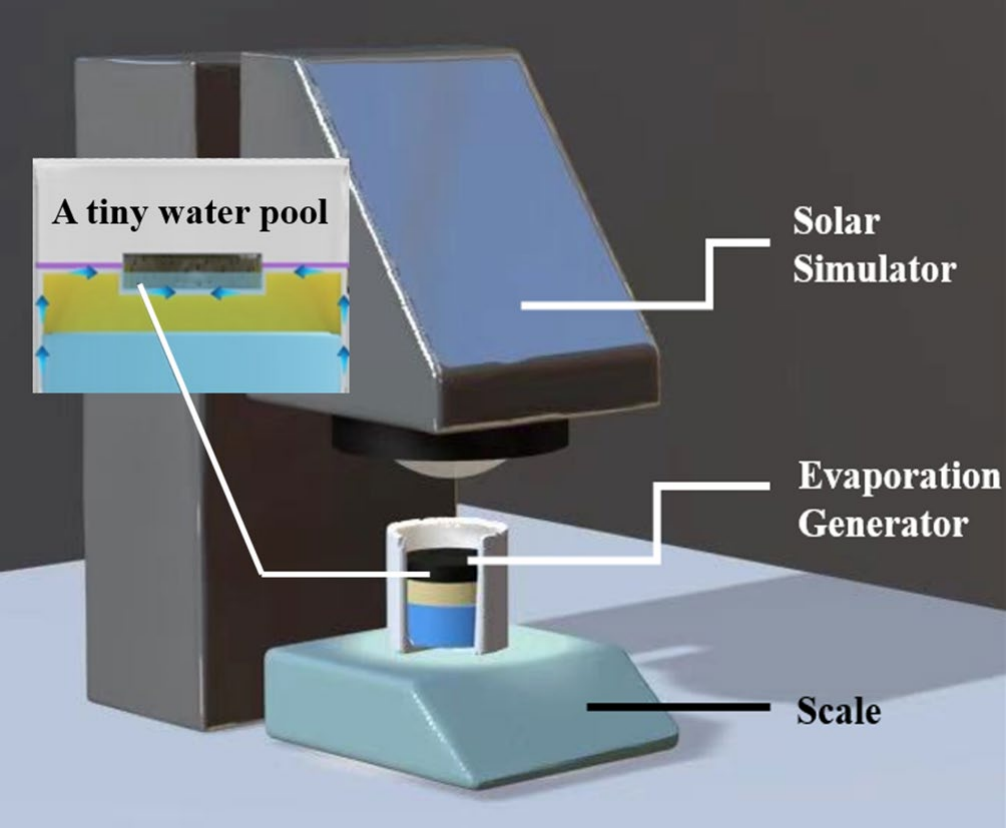
Schematic of solar simulation and solar-driven water desalination system.

A solar simulator lamp in the laboratory is used to simulate one sun illumination (1000 W m^−2^) in the evaporation rate test. Simultaneously, an electronic scale is used to record the evaporation system's mass changes in the real time. The ambient temperature is 25 °C and the humidity is 30%. The simulated environment is shown in Fig. 5.

The reduced water weight in the electronic scale recording is the amount of water evaporated by the light absorber’s photothermal energy to evaporate. This also represents the amount of contaminated water that is purified through this desalination device.

**Figure 6:**
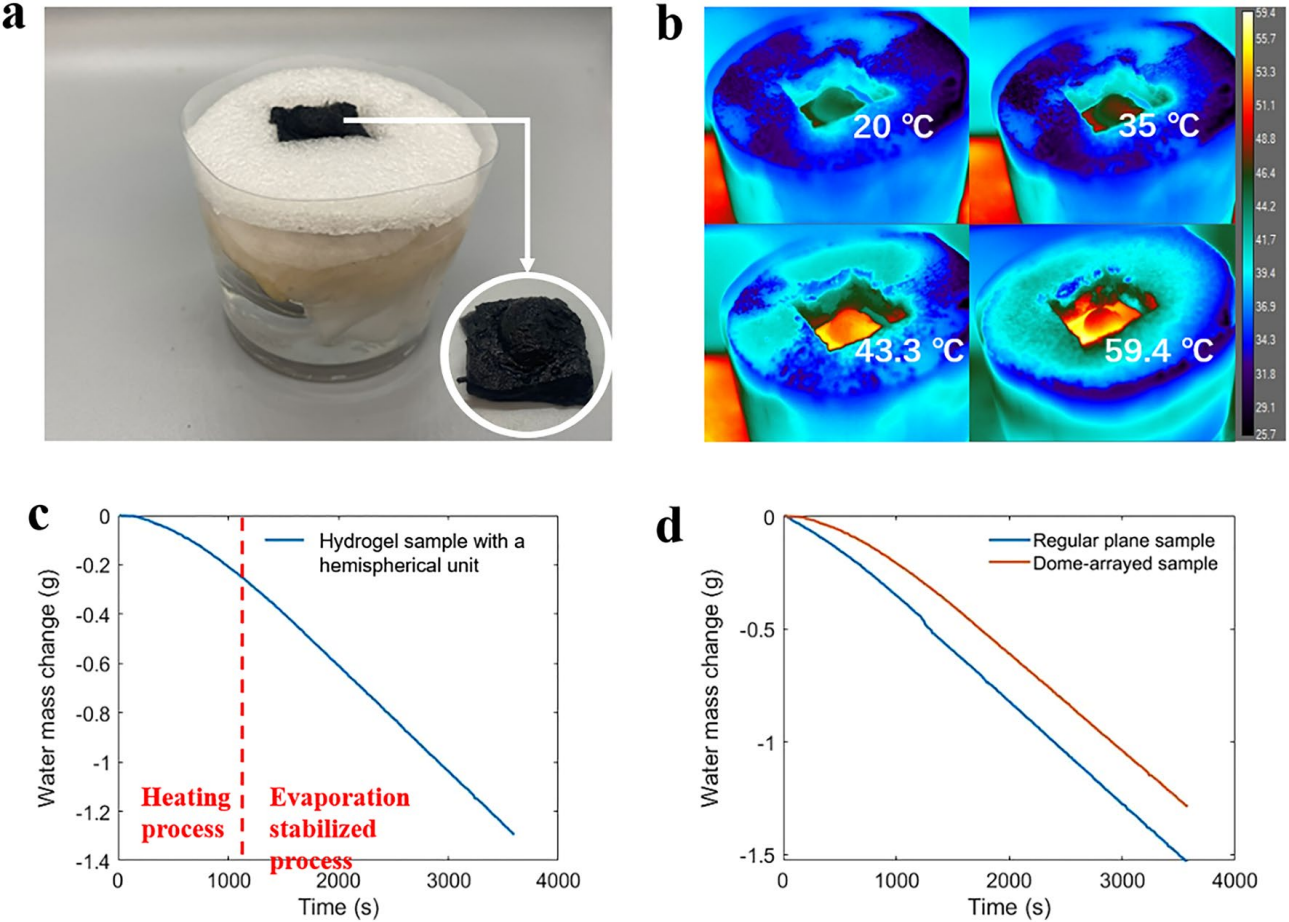
(**a**) Evaporation system with a unit of hemispherical hydrogel sample and the testing unit of hemispherical hydrogel. (**b**) Infrared thermal images of the top surface of hemispherical hydrogel sample during the heating process. (**c**) Water mass change in the system under one sun illumination intensity. (**d**) Water mass changes with dome-arrayed hydrogel sample and with regular plane sample under one sun illumination.

In this experiment, a unit including one hemisphere in the dome-arrayed CPC hydrogel sample is selected as the experimental object. A square sample with a side length of 20 mm is taken from the hydrogel sample. An electric grinder is used to polish its upper and lower surfaces. Simultaneously, an optimized system is demonstrated with a square depression in the middle of heat insulation layer that matched the sample size (Fig. 6a). The sample is placed into the system, and the weight change of system is recorded within one hour under one sun illumination.

During the experiment, an infrared camera is used to record the sample's heating process under light, and the maximum temperature and temperature distribution of the sample surface is studied. The infrared thermal images are shown in Fig. 6b. The initial temperature of the sample is set at room temperature (20 °C). After about 10 min, the temperature reaches stability. At this time, the maximum temperature of the sample surface is 59.4 °C. The temperature at the top of the hemispherical structure is slightly lower than that in the plane part, about 53 °C.

Due to the hemispherical structure, the CPC hydrogel sample thickness is increased compared with that of the plane sample, so the heating process is slower than in previous experiments, and the evaporation efficiency is also affected. The evaporation rate in this process rises as the evaporator approaches a stable temperature, as does the evaporation efficiency. Once the temperature reaches equilibrium, the evaporation rate also reaches a steady state. This change of evaporation rate from low to high due to the temperature change is also reflected in the water weight change curve in Fig. 6c. For this reason, only the data recorded after a relatively stable rate of mass change is reached is considered in the calculation of evaporation rate. As such, the final 30 min of the experiment was chosen to calculate the evaporation rate in the stabilized evaporation process. The evaporation performance of the system is calculated to be 3.80 kg m^−2^ h^−1^ based on a sample surface area of 400 mm^2^ (20 mm × 20 mm).

In a standard double-layer structure, the evaporation behavior of the hydrogels can be used to evaluate the availability of the material for the basic desalination of seawater. Therefore, in this experiment, the efficiency of placing flat cylinder hydrogels in a standard double-layer evaporation system is evaluated. The change in water mass due to evaporation is recorded during a 1-h test under one solar light intensity, as shown in Fig. 6d. The total weight of evaporated water is 1.95 g, and the surface area of the hydrogel sample is 8.55 cm^2^. From this, an evaporation rate of 2.28 kg m^−2^ h^−1^ is determined.

In the optimized evaporation system, the samples' evaporation rate with the dome-arrayed hydrogel is increased by ~ 66.7% with respect to that of regular double-layer system (without dome-arrayed hydrogel and tiny puddle structure). It fully proves that evaporation space is provided for adequate water with the increase of evaporation surface area, and evaporation performance is greatly improved.

**Figure 7:**
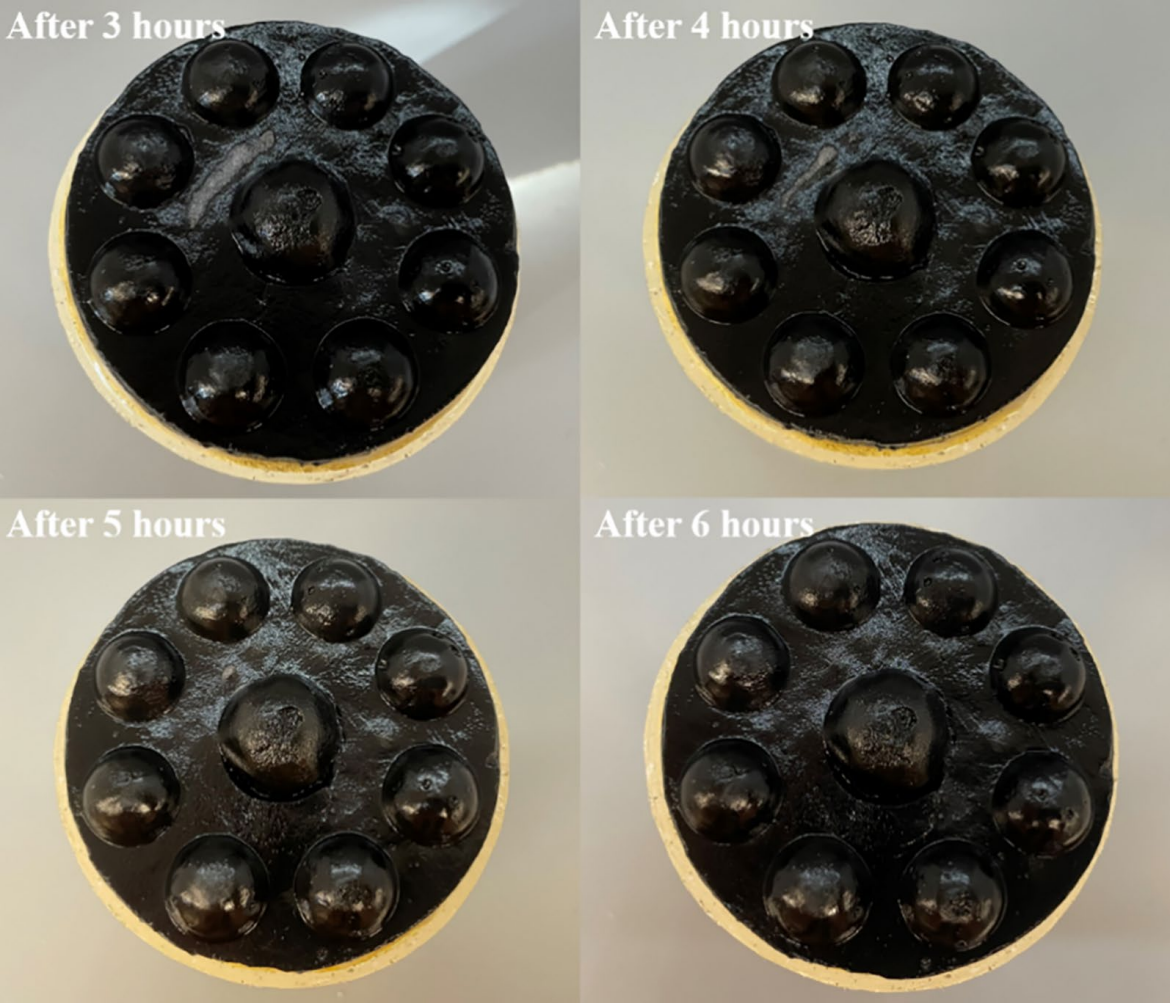
The salt rejection progress of the dome-arrayed chitosan/PVA hydrogel based evaporation device.

In solar evaporation devices, it is necessary to avoid the accumulation of salt during the evaporation process. The dissolution process of salt on the surface of the sample is shown in Fig. 7. It demonstrates the ability of the hydrogel sample to drain salt. Upon contact with water, the solid NaCl on the upper layer starts to dissolve due to the movement and exchange of the solution inside the absorber layer and the water delivery system present on the surface of the device and under the insulation layer. After about 6 h, the triple-layer device completely eliminated the salt, which demonstrates its good salt-removal capacity.

## Conclusion

In this work, a dome-arrayed chitosan/PVA hydrogel based solar-driven water evaporation system has been studied. This system realizes the heating and evaporation of water by leveraging solar energy, resulting in steam generation at a low energy cost and providing a new material for freshwater acquisition. In the system's design, a micro puddle structure based on the double-layer evaporation system is proposed for the first time. A hydrogel sample with a dome-arrayed structure is studied, expanding the structure of the hydrogel from a 2D geometry to a 3D geometry, which provides a larger evaporation area for the evaporator. Meanwhile, the superior light absorption performance of CNPs and the internal pore structure of hydrogel material enable the system to absorb more solar energy and achieve a higher evaporation efficiency through the dome-arrayed structure. By optimizing the geometric parameters, an increase from regular double-layer structure to the tiny puddle structure with a dome arrayed hydrogel absorber in the evaporation rate of ~ 66.7% is realized with respect to the system without 3D hydrogel and puddle. These innovative ideas optimize traditional structures and can result in more efficient and less costly desalination plants, representing a significant leap in renewable energy harvesting and water treatment for developing human societies.

## Data Availability

The authors declare that the main data supporting the findings of this study are contained within the paper. All other relevant data are available from the corresponding author upon reasonable request.
